# New-Onset Graves’ Disease in the Postpartum Period Initially Suspected to Represent Postpartum Thyroiditis

**DOI:** 10.7759/cureus.116275

**Published:** 2026-09-15

**Authors:** Maryam Sharifi, Kasra Navabi

**Affiliations:** 1 Endocrine, Diabetes and Metabolism, Division of General Internal Medicine and Health Services Research, Los Angeles, USA; 2 Medicine/Endocrinology, University of California Los Angeles David Geffen School of Medicine, Los Angeles, USA

**Keywords:** anterior neck pain, graves´disease, postpartum, thyroid-stimulating immunoglobulin (tsi), thyrotropin receptor antibodies (trab)

## Abstract

Postpartum thyroid dysfunction is usually attributed to postpartum thyroiditis (PPT), a self-limited autoimmune condition. Graves' disease (GD) can also develop during the postpartum period, and the two conditions often present with overlapping features. We describe the case of a 37-year-old woman who developed severe anterior neck pain and abnormal thyroid function nine months postpartum, after she stopped breastfeeding. She was initially thought to have PPT, but serial testing showed steadily declining thyroid-stimulating immunoglobulin (TSI) and thyroid-stimulating hormone (TSH) receptor antibody (TRAb) titers, along with increased thyroid vascularity on Doppler ultrasound. These findings ultimately pointed to GD. This report illustrates the importance of longitudinal follow-up and antibody testing when postpartum thyroid dysfunction does not resolve as expected.

## Introduction

Postpartum thyroiditis (PPT) is an autoimmune, destructive thyroiditis occurring within the first year after delivery and is among the most common causes of postpartum thyroid dysfunction. It affects about 5-8% of women, although prevalence varies according to population characteristics, iodine status, thyroid autoantibody status, and the duration of postpartum follow-up [1,2]. The risk is substantially higher in women with thyroid peroxidase (TPO) antibody positivity, type 1 diabetes mellitus or other autoimmune diseases, a personal history of PPT, or a family history of autoimmune thyroid disease [1-3].

The pathophysiology of PPT is related to a rebound of thyroid autoimmunity after the relative immune tolerance of pregnancy resolves. Thyroid autoantibody levels generally decline during pregnancy and rise again postpartum, and renewed immune-mediated destruction of thyroid follicular cells results in the release of preformed thyroid hormone rather than increased thyroid hormone synthesis [1,3,4]. The classic clinical course consists of a transient thyrotoxic phase followed by hypothyroidism and eventual recovery to euthyroidism; however, many patients develop only a thyrotoxic or hypothyroid phase [1,3,5]. The thyrotoxic phase is generally mild and may manifest with palpitations, anxiety, irritability, tremor, fatigue, or weight loss, whereas the hypothyroid phase may cause fatigue, weight gain, constipation, dry skin, impaired concentration, or depressive symptoms [3,5]. Because many of these manifestations overlap with common postpartum symptoms, PPT may go unrecognized. Although thyroid function eventually normalizes in most affected women, some develop persistent hypothyroidism, and recurrence is common in subsequent pregnancies, warranting long-term thyroid surveillance [1,3].

The principal differential diagnosis of postpartum thyrotoxicosis is Graves' disease (GD), although subacute thyroiditis, toxic nodular disease, and exogenous thyroid hormone exposure should also be considered when clinically appropriate [3,5,6]. Differentiating PPT from GD is particularly important because their pathophysiology, treatment, prognosis, and implications for subsequent pregnancies differ. Serum thyroid-stimulating hormone (TSH) and free thyroxine (FT4) establish the presence and severity of thyroid dysfunction, while TPO antibodies support underlying thyroid autoimmunity [1,3,5]. Thyrotropin receptor antibodies (TRAb), including thyroid-stimulating immunoglobulins (TSI), are typically positive in GD and generally absent in PPT [1,3,6,7]. Radioactive iodine uptake is low in destructive thyroiditis and normal or increased in GD, although breastfeeding may limit its use in the postpartum period [3,5-7]. Thyroid ultrasonography with color Doppler may provide additional supportive evidence, with reduced vascularity favoring destructive thyroiditis and increased vascularity favoring GD [7,8].

Management depends on the phase and severity of PPT. Because thyrotoxicosis results from the release of preformed thyroid hormone rather than increased hormone synthesis, antithyroid medications are not effective; symptomatic patients may instead be treated with beta-adrenergic blockade [1,3,5]. Levothyroxine may be indicated during the hypothyroid phase in patients with significant symptoms, marked biochemical hypothyroidism, or plans for pregnancy, with subsequent reassessment to determine whether hypothyroidism is permanent [1,3]. Important complications include persistent hypothyroidism and recurrence of PPT following subsequent pregnancies [1,3].

GD may also develop or recur during the postpartum period as autoimmune activity rebounds following pregnancy [4,9]. Postpartum GD typically develops later than PPT, and the postpartum increase in autoimmune activity can make early clinical differentiation challenging [3,4,9]. Here, we describe a patient whose postpartum GD was initially attributed to PPT because of the timing of presentation and the presence of anterior neck pain, despite an unusual biochemical evolution and persistent thyroid-stimulating antibodies. This report highlights the importance of serial biochemical and antibody assessment when the clinical course does not follow the expected pattern of PPT.

## Case presentation

A 37-year-old woman, gravida 1 para 1, was referred for an evaluation of thyroid dysfunction at nine months postpartum after the birth of her first child. She underwent a cesarean delivery at Month zero and discontinued breastfeeding at Month nine postpartum. Around this time, she developed severe anterior neck pain, initially thought to be a sore throat. Laboratory testing revealed an elevated TSH, and PPT was initially considered the most likely diagnosis. She had also suffered from postpartum depression in the months following delivery, which had gradually resolved. Her family history was significant for hypothyroidism in her mother and thyroid disease in three paternal aunts.

Repeat thyroid testing conducted over the following months revealed substantial variation in thyroid function. Serial thyroid function tests and thyroid autoantibody titers are presented in Table 1. Thyroid ultrasonography revealed a mildly enlarged gland with heterogeneous parenchyma and increased vascularity on color Doppler imaging (Figure 1).

**Table 1:** Serial thyroid function tests and thyroid autoantibodies TSH: thyroid-stimulating hormone; Free T4: free thyroxine; TRAb: TSH receptor antibody; TSI: thyroid-stimulating immunoglobulin

Months postpartum	TSH (mIU/L)	Free T4 (ng/dL)	TRAb (IU/L)	TSI (IU/L)
9	41.9	0.4		5.39
10	0.02	2.3		4.98
13	2	1	3.11	3.7
16	2.8		1.79	1.72
18	2.6	1.2	1.18	0.71
Reference range	0.3-4.7	0.80-1.7	≤ 1.75	≤ 0.55

**Figure 1:**
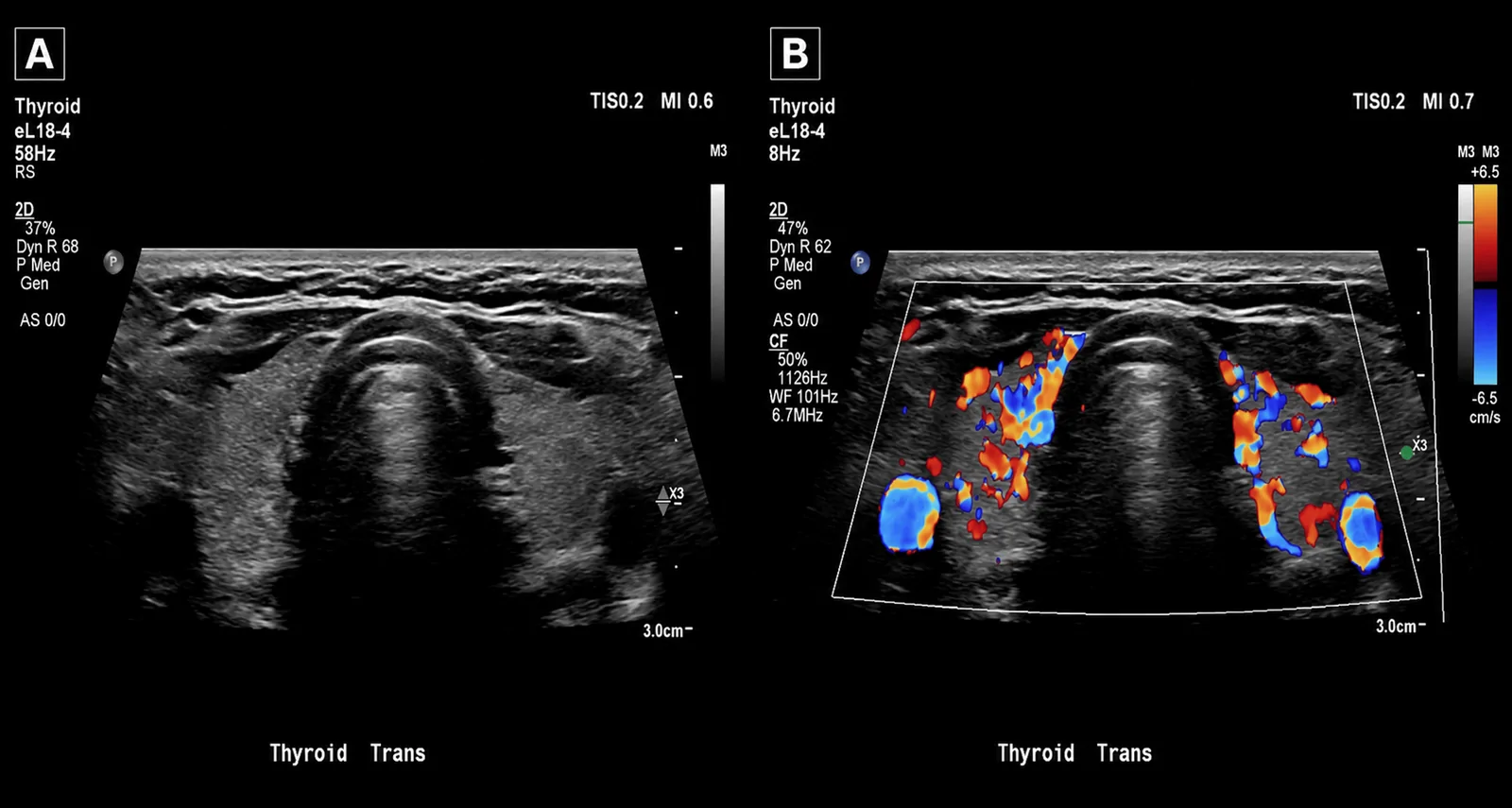
Thyroid ultrasound without and with Doppler (A) Transverse grayscale ultrasound image of the thyroid gland without Doppler. (B) Transverse color Doppler ultrasound image demonstrating thyroid vascularity

At Month nine postpartum, the patient presented with overt hypothyroidism, with a TSH of 41.9 mIU/L and free T4 of 0.4 ng/dL. TSI was elevated at 5.39 IU/L. By Month 10, the biochemical pattern had shifted to overt hyperthyroidism, with a TSH of 0.02 mIU/L and free T4 of 2.3 ng/dL; TSI remained elevated at 4.98 IU/L. At Month 13, thyroid function had normalized, although TRAb and TSI remained elevated at 3.11 IU/L and 3.7 IU/L, respectively. Antibody titers subsequently declined, with TRAb falling to 1.79 IU/L and TSI to 1.72 IU/L at Month 16. By Month 18, TRAb had fallen to 1.18 IU/L and TSI to 0.71 IU/L, with TSH and free T4 remaining within the reference range.

The combination of persistent thyroid-stimulating antibodies, the transition from hypothyroidism to hyperthyroidism, and increased thyroid vascularity raised suspicion for Graves' disease rather than isolated postpartum thyroiditis. Methimazole 5 mg daily was started, leading to subsequent symptomatic and biochemical improvement. Serial measurements showed progressive reduction in both TRAb and TSI during treatment.

The patient also voiced interest in a future pregnancy. Counseling addressed the importance of achieving stable euthyroidism before conception and monitoring thyroid receptor antibodies given their relevance during pregnancy. At the most recent assessment at Month 18, thyroid function was normal, and both TRAb and TSI had substantially declined.

## Discussion

Pregnancy is characterized by relative immune tolerance, followed by a rebound in immune activity during the postpartum period that can precipitate or exacerbate autoimmune thyroid disease in susceptible women [3,4]. PPT is considerably more common than postpartum GD, affecting approximately 5-8% of women following delivery [2,3]. Nevertheless, GD should remain an important consideration when postpartum thyroid dysfunction persists, thyroid-stimulating antibodies are present, or the clinical course does not follow the expected pattern of destructive thyroiditis [1,3]. Key clinical and diagnostic features distinguishing PPT from postpartum GD are summarized in Table 2.

**Table 2:** Distinguishing features of PPT versus postpartum GD GD: Graves' disease; PPT: postpartum thyroiditis; T3: triiodothyronine; T4: thyroxine; TRAb: thyrotropin receptor antibody; TSI: thyroid-stimulating immunoglobulin

Feature	PPT	Postpartum GD	References
Typical onset	1–6 months postpartum	4–12 months postpartum (peak 7–9 months)	[1,3,4]
Neck pain	Painless (anterior neck pain atypical for both; suggests subacute thyroiditis)	Painless (anterior neck pain atypical for both; suggests subacute thyroiditis)	[5]
TRAb/TSI	Usually negative	Positive	[1,3,6,7]
Total T3:T4 ratio	Low (< 20)	High (> 20)	[6,7]
Thyroid Doppler vascularity	Low or absent	Diffusely increased	[6-8]
Radioactive iodine uptake	Low	Elevated or normal	[3,5-7]
Goiter	Mild or absent	Often prominent, may have bruit	[6,7]
Ophthalmopathy	Absent	May be present	[6,7]
Clinical course	Self-limited (weeks to months)	Persistent without treatment	[3,5,7]
Treatment	Supportive (beta-blockers if needed)	Antithyroid drugs	[1,3,5,7]

Timing can also assist with differentiation, since PPT generally presents earlier in the postpartum period, whereas postpartum GD tends to appear later [3,4]. Our patient's presentation at nine months postpartum therefore remained consistent with postpartum GD despite the initial suspicion for PPT. Her initial presentation was unusual because several features suggested thyroiditis. The patient had severe anterior neck pain, even though PPT is characteristically painless. Prominent thyroid pain is more typical of subacute (de Quervain) thyroiditis and should prompt consideration of alternative or overlapping causes [5]. Additionally, she initially presented with overt hypothyroidism, with a TSH of 41.9 mIU/L and free T4 of 0.4 ng/dL, a biochemical pattern that could reasonably suggest the hypothyroid phase of PPT. However, one month later she developed overt hyperthyroidism, and thyroid-stimulating antibodies remained markedly elevated. This progression was difficult to reconcile with isolated PPT.

The sequence of thyroid dysfunction was particularly informative. The classic course of PPT consists of an initial thyrotoxic phase resulting from the release of preformed thyroid hormone, followed by a hypothyroid phase and eventual recovery, although many patients experience only one phase [3,5]. In this patient, the reverse sequence, marked hypothyroidism followed by hyperthyroidism, was atypical for classic PPT. The persistent TSI elevation during the hyperthyroid phase and subsequent TRAb positivity favored the emergence of GD, possibly superimposed on an initial destructive thyroiditis process. The 2026 ATA Guidelines emphasize the diagnostic value of TRAb and TSI in distinguishing GD from destructive postpartum thyroid dysfunction, as thyroid-stimulating antibodies are generally present in GD and absent in PPT [1].

Ultrasonography provided additional supportive evidence. Color Doppler imaging revealed increased thyroid vascularity, a finding more characteristic of GD than of destructive thyroiditis. Doppler ultrasonography may be particularly useful when radioactive iodine uptake testing is undesirable or impractical, such as during lactation [3,6-8]. Increased diffuse vascularity reflects the increased thyroid blood flow associated with GD, whereas destructive thyroiditis generally shows lower vascularity [6-8]. In this case, the qualitative Doppler findings, taken together with persistent thyroid-stimulating antibodies and the evolving biochemical pattern, provided stronger support for GD than for isolated PPT.

Correctly distinguishing GD from PPT is clinically important because their management approaches differ substantially. Thyrotoxicosis caused by PPT results from the release of preformed thyroid hormone and therefore does not respond to antithyroid medications; treatment is generally supportive, with beta-adrenergic blockade when clinically indicated [3,5]. GD, by contrast, results from increased thyroid hormone synthesis and is appropriately treated with antithyroid medication [6,7]. The patient's subsequent clinical and biochemical response to low-dose methimazole further confirmed the diagnosis of GD.

Serial antibody measurements also provided useful insight into disease evolution. TRAb declined from 3.11 IU/L at Month 13 to 1.79 IU/L at Month 16 and then to 1.18 IU/L at Month 18, eventually falling within the laboratory reference range. TSI likewise declined from 3.70 IU/L at Month 13 to 1.72 IU/L at Month 16 and then to 0.71 IU/L at Month 18, though it remained mildly above the laboratory upper limit of normal of 0.55 IU/L. This progressive decline paralleled the normalization of thyroid function and suggested decreasing autoimmune activity.

Prognosis of postpartum Graves' disease

Postpartum GD may respond more favorably to antithyroid therapy than non-postpartum relapsing GD. Rotondi et al. reported a significantly higher remission rate following a second course of methimazole among women with postpartum relapse compared to women whose relapse was unrelated to pregnancy (79% versus 32%, respectively; p = 0.002). The postpartum group also showed a more rapid decline in TRAb concentrations [9]. These findings support the concept that postpartum immune rebound may be transient in some patients and that a conservative antithyroid-drug approach can be appropriate when clinically feasible.

Our patient's course was consistent with this favorable pattern. Thyroid function normalized, TRAb returned to the reference range, and TSI decreased substantially during follow-up. Because TSI remained mildly elevated at Month 18, however, continued biochemical and antibody surveillance remains appropriate, particularly for future pregnancy planning.

Implications for future pregnancy

The patient's desire for another pregnancy has important implications for management. Maternal TRAb can cross the placenta and stimulate the fetal thyroid, creating a risk of fetal and neonatal hyperthyroidism. Current guidelines therefore emphasize achieving stable euthyroidism before conception and monitoring TRAb in women with current or previous GD [1,10].

The 2026 ATA Guidelines recommend that women with hyperthyroidism who are planning pregnancy achieve euthyroidism before conception and undergo appropriate preconception counseling regarding antithyroid treatment [1]. TRAb measurement is particularly important because sufficiently elevated maternal antibody concentrations are associated with fetal and neonatal thyroid dysfunction. During pregnancy, women with elevated TRAb or with a need for antithyroid medication may require repeat antibody measurement and fetal surveillance [1]. The 2017 ATA Pregnancy Guidelines similarly recommend early pregnancy TRAb measurement in women with a history of GD treated with radioactive iodine or thyroidectomy, followed by repeat measurement later in pregnancy when levels remain elevated [10].

Antithyroid drug selection also requires special consideration around conception and early pregnancy. Because methimazole exposure during organogenesis is associated with a characteristic pattern of congenital malformations, propylthiouracil is generally preferred during the first trimester. Consideration may then be given to switching to methimazole after the first trimester due to the risk of severe hepatotoxicity associated with prolonged propylthiouracil exposure [1,11].

For this patient, the progressive decline in TRAb and TSI is reassuring; however, the Month 18 TSI remained slightly above the laboratory reference range. Therefore, thyroid function and thyroid receptor antibody status should be reassessed before conception instead of assuming complete immunologic remission based solely on euthyroid thyroid function.

Postpartum depression and thyroid dysfunction

Thyroid dysfunction and postpartum depressive symptoms can coexist and may share nonspecific clinical manifestations such as fatigue, impaired concentration, sleep disturbance, and weight changes. However, current evidence has not established a consistent causal link between PPT or thyroid autoimmunity and postpartum depression [1,3]. Thyroid function testing remains appropriate when symptoms or clinical circumstances raise concern about thyroid dysfunction, because biochemical thyroid disease may otherwise be mistaken for symptoms commonly attributed to the postpartum state.

In this patient, postpartum depression improved gradually, while the thyroid abnormalities followed an independent and prolonged course of their own. Although a causal relationship cannot be established, the coexistence of these conditions reinforces the importance of considering thyroid dysfunction when evaluating persistent or atypical postpartum symptoms.

Long-term monitoring

Long-term follow-up is warranted after postpartum autoimmune thyroid disease. Women who develop PPT remain at increased risk of recurrent PPT in subsequent pregnancies and of permanent hypothyroidism later in life [1-3]. Periodic thyroid function assessment is therefore recommended even after apparent recovery. Patients with GD also require continued surveillance after treatment because biochemical remission does not necessarily imply permanent immunologic remission. In the present case, normalization of TRAb alongside persistent mild TSI elevation at Month 18 supports continued monitoring. This is particularly important before and during any future pregnancy, when the recurrence of maternal hyperthyroidism and transplacental passage of thyroid-stimulating antibodies could affect both maternal and fetal thyroid function.

Overall, this case illustrates the diagnostic difficulty that can arise when postpartum autoimmune thyroid disorders overlap in timing and clinical presentation. The initial hypothyroidism and postpartum context supported consideration of PPT, while the subsequent development of hyperthyroidism, persistent thyroid-stimulating antibodies, increased Doppler vascularity, and response to methimazole favored postpartum GD. Serial thyroid function testing, TRAb and TSI measurements, and qualitative Doppler ultrasonography proved to be the most useful diagnostic tools in establishing the final diagnosis and guiding management.

## Conclusions

This patient's presentation initially appeared consistent with PPT, given the timing of onset and the neck pain, but her antibody profile, ultrasound findings, and clinical course over time were ultimately more consistent with GD. The case underscores several practical points for clinicians evaluating postpartum thyroid dysfunction. Timing of onset may provide an important diagnostic clue, as GD tends to present later in the postpartum period than PPT. TRAb and TSI testing are particularly useful when postpartum thyroid dysfunction does not follow the expected self-limited course, and persistent thyroid-stimulating antibody positivity strongly supports GD. When radioactive iodine uptake testing is undesirable or impractical during lactation, color Doppler ultrasonography can provide additional supportive information, with diffusely increased thyroid vascularity favoring GD over destructive thyroiditis.

Serial antibody monitoring can also help in assessing disease evolution and response to treatment. In this patient, TRAb and TSI declined substantially over five months of methimazole therapy, with TRAb normalizing and TSI approaching the reference range. For women with postpartum GD who desire future pregnancy, preconception counseling should emphasize achieving stable euthyroidism before conception, reassessing TRAb and TSI, and planning appropriate antithyroid drug management during early pregnancy. Postpartum GD may follow a more favorable course than non-postpartum relapsing GD in some patients, supporting continued antithyroid drug therapy and careful monitoring rather than premature definitive treatment when clinically appropriate.
